# Clot Degradation Under the Action of Histotripsy Bubble Activity and a Lytic Drug

**DOI:** 10.1109/TUFFC.2021.3052393

**Published:** 2021-08-27

**Authors:** Samuel A. Hendley, Jonathan D. Paul, Adam D. Maxwell, Kevin J. Haworth, Christy K. Holland, Kenneth B. Bader

**Affiliations:** Committee on Medical Physics, University of Chicago, Chicago, IL 60637 USA; Department of Medicine, Cardiology Section, University of Chicago, Chicago, IL 60637 USA.; Department of Urology, University of Washington, Seattle, WA 98105 USA.; Department of Internal Medicine, Division of Cardiovascular Health and Disease, and Biomedical Engineering, University of Cincinnati, Cincinnati, OH 45267 USA.; Department of Internal Medicine, Division of Cardiovascular Health and Disease, and Biomedical Engineering, University of Cincinnati, Cincinnati, OH 45267 USA.; Radiology Department and the Committee on Medical Physics, University of Chicago, Chicago, IL 60637 USA.

**Keywords:** Biomedical acoustics, cavitation, fibrinolysis, hemolysis, histotripsy

## Abstract

Deep vein thrombosis is a major source of morbidity worldwide. For critical obstructions, catheter-directed thrombolytics are the frontline therapy to achieve vessel recanalization. Techniques that aid lytic therapy are under development to improve treatment efficacy and reduce procedure-related complications. Histotripsy is one such adjuvant under development that relies on focused ultrasound for *in situ* nucleation of bubble clouds. Prior studies have demonstrated synergistic effects for clot dissolution when histotripsy is combined with lytic therapy. The success of this combination approach is hypothesized to promote thrombolytic efficacy via two mechanisms: erythrocyte fractionation (hemolysis) and increased lytic activity (fibrinolysis). In this study, the contributions of hemolysis and fibrinolysis to clot degradation under histotripsy and a lytic were quantified with measurements of hemoglobin and D-dimer, respectively. A linear regression analysis was used to determine the relationship between hemoglobin, D-dimer, and the overall treatment efficacy (clot mass loss). A similar analysis was conducted to gauge the role of bubble activity, which was assessed with passive cavitation imaging, on hemolysis and fibrinolysis. Tabulation of these data demonstrated hemolysis and fibrinolysis contributed equally to clot mass loss. Furthermore, bubble cloud activity promoted the generation of hemoglobin and D-dimer in equal proportion. These studies indicate a multifactorial process for clot degradation under the action of histotripsy and a lytic therapy.

## I. Introduction

DEEP vein thrombosis is a prevalent condition that afflicts approximately 5% of the population in the United States [1]. Up to 50% of deep vein thrombosis cases develop comorbidities, such as chronic venous insufficiency and postthrombotic syndrome [2]. For most venous obstructions, noninvasive approaches, such as anticoagulation therapy and compression socks, are prescribed to mitigate thrombus growth and restore blood flow [1], [3]. Though these prophylactic techniques are useful for low-risk patients, more aggressive approaches are required for iliofemoral thrombi, which oftentimes are a primary source of pulmonary embolism [4]. Embolization of thrombus to the pulmonary artery carries a high risk of long-term morbidity (e.g., venous insufficiency and pulmonary hypertension) and a 30% mortality rate [5], [6]. To achieve vessel recanalization, catheter-directed recombinant tissue plasminogen activator (rt-PA) is the primary treatment for iliofemoral deep vein thrombosis and other thrombo-occlusive disease in the United States [2]. The lytic rt-PA promotes the enzymatic reduction of fibrin, the extracellular scaffolding for acute thrombus, to D-dimer protein fragments and other fibrin degradation products [7]-[9]. Limitations of this approach include the length of hospitalization time [2], risk of bleeding complications [10], and a limited lytic response for mature thrombus [11].

Adjuvant mechanical therapies are under development to overcome the limitations of catheter-directed lytics. One such approach is sonothrombolysis, which is the application of ultrasound energy to promote the efficacy of lytic therapy. Sonothrombolysis has been shown to be effective in preclinical and clinical studies. Though there are a number of potential mechanisms by which ultrasound acts to improve the efficacy of rt-PA [12]-[14], acoustic cavitation is the primary catalyst for sonothrombolysis [15].

Bubble activity can also be used to break up clots in the absence of a lytic [15]. One means to generate strong bubble activity on demand is histotripsy, a focused ultrasound therapy under development to ablate thrombi [16] among other pathological conditions [17]-[19]. Histotripsy sources generate high tensile pulse amplitudes to activate intrinsic nanoscale nuclei that permeate clots [20], [21]. Growth of the nanoscale nuclei into bubbles 10–1000 *μ*m in diameter imparts strain resulting in hemolysis, disruption of the fibrin network [22]-[24], and restoration of flow.

There are advantages to combining the enhanced fibrinolytic effect of sonothrombolysis approaches with the mechanical destruction caused by histotripsy insonation schemes. Sonothrombolysis approaches have limited efficacy for clots nonresponsive to lytic [25]. In the absence of lytic, histotripsy bubble activity may undertreat clot near the vessel wall [26]. Combining histotripsy with a lytic therapy may therefore provide an effective means to promote clot degradation. Preclinical studies have demonstrated that histotripsy combined with lytic significantly enhances overall treatment efficacy [15], [24]. Two primary mechanisms are hypothesized to contribute to combined histotripsy and lytic therapy: 1) hemolysis due to the mechanical strain of bubble cloud expansion within the clot [22], [27] and 2) enhanced fibrinolysis caused by increased penetration of lytic into the thrombus due to vigorous bubble cloud oscillations [28].

The aim of this study was to gauge the contributions of hemolysis and fibrinolysis to the overall treatment efficacy for the combination therapy. Clots were exposed to rt-PA and histotripsy pulses using a venous flow model, as described previously [24]. A transducer designed for the treatment of deep vein thrombosis was used to deliver histotripsy pulses. The range of insonation conditions (i.e., peak negative pressure and pulse duration) spans those explored in other histotripsy clot ablation studies [24], [26]. The percent change in clot mass was used as the overall metric of treatment efficacy. Assays to measure hemoglobin and D-dimer within the perfusate following histotripsy and lytic exposure were used to quantify the contribution of mechanical and fibrinolytic effects, respectively. A secondary aim of this study was to determine the role of bubble activity in promoting hemolysis or fibrinolysis. To quantify bubble activity, passive cavitation imaging was used to map the pressure generated by histotripsy bubble cloud activity [29]. Linear regression analysis was conducted to assess the relationship between hemolysis, fibrinolysis, and overall treatment efficacy. Additional linear regression analysis was conducted to compare the effect of bubble activity on mechanical hemolysis and chemical fibrinolysis. Findings were also compared to the gross histological observation of the histotripsy zone in the clot.

## II. Methods

### A. Venous Whole-Blood Clot Model

Human whole-blood clots were manufactured following an established Internal Review Board-approved protocol (University of Chicago IRB #19-1300) [24], [30]. Briefly, venous human whole blood was drawn from 11 volunteer patients undergoing invasive catheterization procedures at the University of Chicago Medicine Cardiac Catheterization Laboratory following informed consent. Aliquots of blood (2-mL volume) were transferred to borosilicate Pasteur pipettes (Fisher Scientific, Pittsburg, PA, USA), heated at 37 °C for 3 h, stored for a minimum of three days at 4 °C to ensure full retraction [31], and used within two weeks.

### B. Preparation of Recombinant Tissue-Type Plasminogen Activator and Human Fresh-Frozen Plasma

Lytic (rt-PA) was obtained from the manufacturer (Activase, Genetech, San Francisco, CA, USA) as lyophilized powder. Each vial was mixed with sterile water to a concentration of 1 mg/mL, aliquoted into 0.5-mL centrifuge tubes, and stored at −80 °C. Aliquots were thawed within three years of freezing and used within 4 h of thawing for each experiment. When stored in this manner, rt-PA is stable over the course of at least seven years [32]. Human fresh-frozen-type O plasma was procured from a blood bank (Vitalant, Chicago, IL, USA), aliquoted, and stored at −80 °C prior to use.

### C. Histotripsy Insonation

Histotripsy pulses were generated by an eight-element, elliptically focused transducer (9-cm major axis, 7-cm minor axis, and 6-cm focal length) [33]. The fundamental frequency of the transducer was 1.5 MHz, and the resultant focal zone under typical driving conditions had a −6-dB focal width of 4.27 mm × 0.66 mm × 0.70 mm for the axial direction, and the major and minor axes, respectively, relative to the transducer. An L11-5v imaging array (Verasonics, Inc., Kirkland, WA, USA) was placed through a coaxial opening on the histotripsy transducer to provide image guidance using a research ultrasound scanner (Vantage 128, Verasonics, Inc., Kirkland, WA, USA). The histotripsy transducer elements were excited in parallel by a custom-designed and built class D amplifier and matching network [34], [35].

### D. Experimental Procedure

**Fig. 1.**
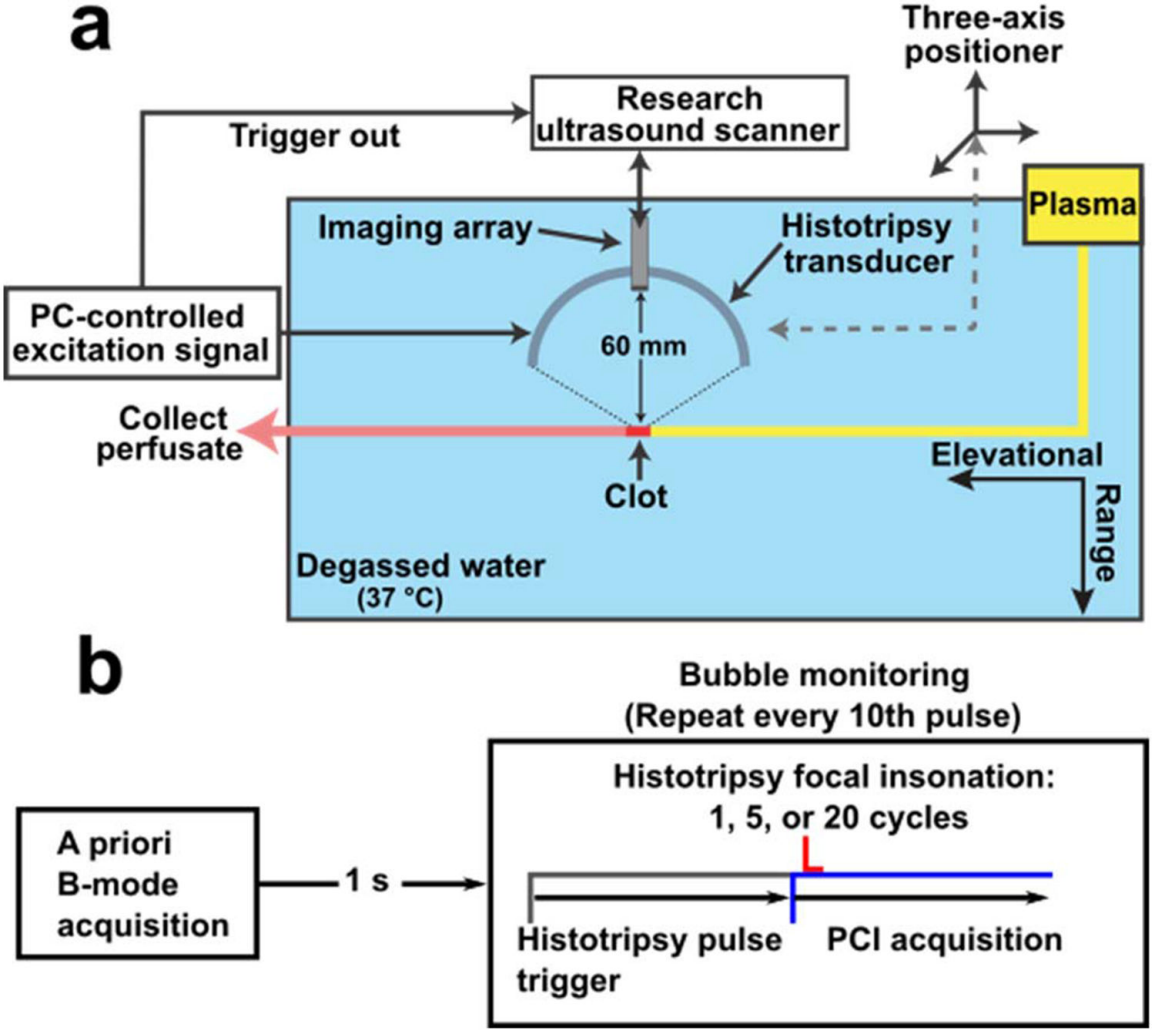
Experimental setup. (a) Schematic of the flow channel setup. The flow was from right to left in this diagram. The histotripsy source moves along the direction of flow. (b) Timing diagram for histotripsy insonation and image data acquisition. To identify the clot location, a B-Mode ultrasound image was taken prior to the application of the histotripsy therapy. During the application of histotripsy, acoustic emissions generated by the bubble could were acquired passively and beamformed to generate passive cavitation images (PCI) offline.

Prior to histotripsy exposure, the clot was cut to 1 cm in length and introduced into a venous flow model [24] (see Fig. 1). The model was composed of a syringe pump (EW-74900-20, Cole-Parmer, Vernon Hills, IL, USA) that drew plasma through 6.35-mm-inner-diameter and 0.79-mm wall thickness latex tubing (McMaster-Carr, Elmhurst, IL, USA) at a rate of 0.65 mL/min. This flow rate was chosen to mimic the flow observed for a partially occluded iliofemoral vessel [36]. The plasma was maintained at physiologic temperature by submerging it in a bath of degassed (20% dissolved oxygen), reverse osmosis water heated to 37.3 °C ± 0.5 °C. The flow channel was perfused with plasma alone or plasma and rt-PA (2.68 *μ*g/mL) 5 min before insonation began [37].

Translation of the histotripsy source along the length of the clot was achieved with three orthogonal motorized linear stages (BiSlide, Velmex Inc., Bloomfield, NY, USA). The clot was targeted as described previously [16], [24]. Briefly, the location of the transducer focus in the plane of the coaxial imaging array was determined by generating histotripsy pulses in the degassed water outside the lumen. The bubble cloud appeared hyperechoic in the imaging plane as described previously [16], [24], [38]. Once the location of the focus was determined, the transducer was aligned with the distal end of the clot relative to the pump (far right, Fig. 1). Waypoints (i.e., positions of the motors where the bubble cloud was contained within the clot) were set every 5 mm along the length of the clot. A custom script interpolated an automated path for the transducer along the length of the clot with a 0.5 mm step size (~20 steps per clot). The step-size distance was smaller than the −6-dB width of the focal zone along the dimension of transducer translation. At each location, 2000 pulses were applied at a 40 Hz rate, consistent with other thrombotripsy studies [15], [24], [39]. The total treatment time was 25 min/clot. The insonation pulse had a duration of 1, 5, or 20 cycles (0.67, 3.33, or 13.33 *μ*s, respectively). For one-cycle pulses, peak negative pressures of 0, 25, 30, or 35 MPa were explored. A 35-MPa peak negative pressure was used for 5 and 20 cycle arms. A minimum of eight clots were used for each treatment arm (122 total).

### E. Imaging Assessment of Bubble Activity

**Fig. 2.**
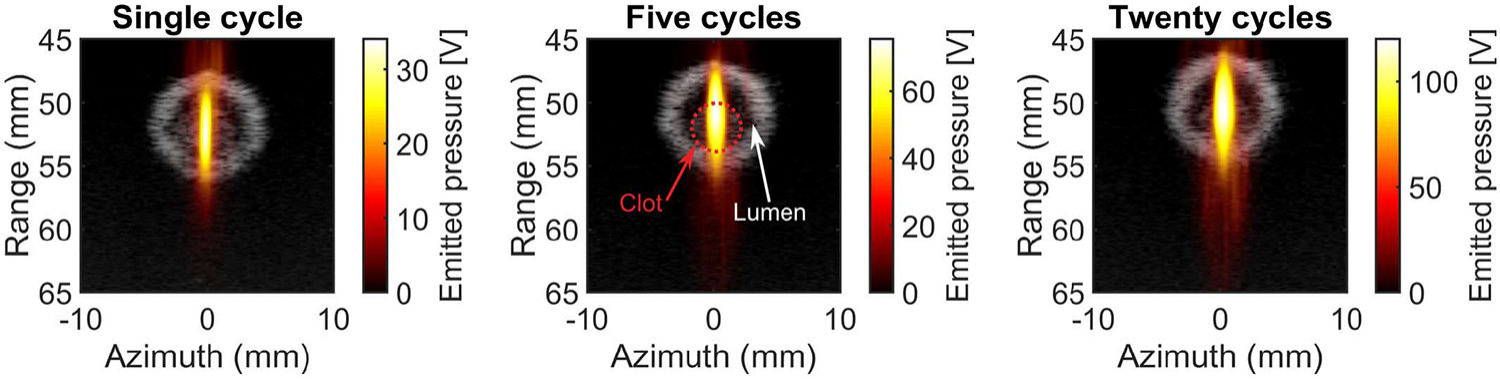
Duplex B-mode overlaid with passive cavitation data for single-, five-, and twenty-cycle pulses (35-MPa peak negative pressure). In each image, the histotripsy pulse propagates from the top to the bottom. Although the focal length of the histotripsy transducer is 60 mm, the imaging array protrudes slightly beyond the opening of the transducer. This positioning of the imaging array explains why the focus appears before 60 mm relative to the imaging array.

At each insonation location, a B-mode ultrasound image was acquired prior to histotripsy exposure and segmented manually to denote the area of the clot (see Fig. 2). During the insonation, acoustic emissions from the bubble cloud were acquired passively with the array and processed offline to form passive cavitation images (PCIs) using the robust Capon beam-former [40]. Because of data transfer rate limitations, acoustic emissions were acquired once every ten histotripsy pulses (200 frames per location). All 200 frames were averaged pixelwise to generate a cumulative image for each treatment location (20 per clot). To calculate the total emitted pressure within the clot, the cumulative PCI was coregistered with the B-mode image and summed spatially over the clot area

(1) $$P_{\text{clot}}=\sum_{i=1}^{21}\sum_{r}^{\forall r\in clot}P_{i}(r)$$

where *P_i_* (*r*) is the PCI emitted pressure at location *r* for a treatment location *i* [24].

### F. Quantification of Hemolysis and Fibrinolysis

Within 1 min of the end of treatment, the lumen was drained and the perfusate was transferred to microcentrifuge tubes for subsequent analysis. One end of the lumen was opened and tilted to allow for the extraction of the remaining clot segment via gravity. Clot mass loss was measured using a digital balance before and after treatment blotting the clot before measurement to remove excess fluid [15], [24], [31], [41]. The concentration of hemoglobin and D-dimer in the perfusate was assayed to gauge hemolysis and fibrinolysis, respectively. Perfusate samples were stored at 4 °C for no more than 4 h between sample collection and subsequent analysis. Preliminary studies indicated that samples were stable over this period, with no significant change in the estimated hemoglobin or D-dimer. Drabkin’s reagent assay (Sigma-Aldrich, St. Louis, MO, USA) was used to quantify hemoglobin, a marker of hemolysis [42]. A 1-mL sample of the perfusate was centrifuged at 610 RCF (3500 RPM) for 10 min, after which the supernatant and Drabkin’s reagent were combined in a one-to-one ratio (0.5 mL each). The optical absorbance of the solution was measured at 540 nm using a plate reader (Synergy Neo HST, BioTek, Winooski, VT, USA), which was converted to hemoglobin concentration using a standard curve. An additional 1-mL sample of the perfusate was mixed with 0.1 mg/*μ*L of 6-aminocaproic acid (Sigma-Aldrich, St. Louis, MO, USA) to halt fibrinolysis and was subjected to a latex immuno-turbidimetric assay (STA R Max, Stago, Asnières-sur-Seine, France) to quantify the D-dimer molecule concentration, a marker of fibrin degradation by rt-PA [7]-[9]. To examine structural changes, selected clots were fixed in formalin, embedded in paraffin, sectioned, stained with Hematoxylin & Eosin, and digitally scanned.

### G. Statistical Analysis

Statistical analysis was performed using MATLAB 2017b (The Mathworks, Natick, MA, USA). A one-way analysis of variance (ANOVA) test was performed in conjunction with Tukey’s honest significant difference test using an *α* level of 0.05 to determine differences in mass loss, hemoglobin concentration, or D-dimer concentration between arms. Linear regression coefficients, reported as *β* values, were calculated to quantify the relationship between hemoglobin or D-dimer and mass loss. Linear regression coefficients were also calculated to determine the relationship between PCI pixel values and hemoglobin or D-dimer (62). Confidence intervals of the *β*-values (*α* = 0.05) were calculated by the Wald method (63). A similar analysis was performed to compare the effect of bubble activity on hemoglobin and D-dimer concentration. Differences in D-dimer concentrations between lytic and non-lytic arms were analyzed using a two-tailed paired Student’s t-test.

## III. Results

### A. Treatment Efficacy

**Fig. 3.**
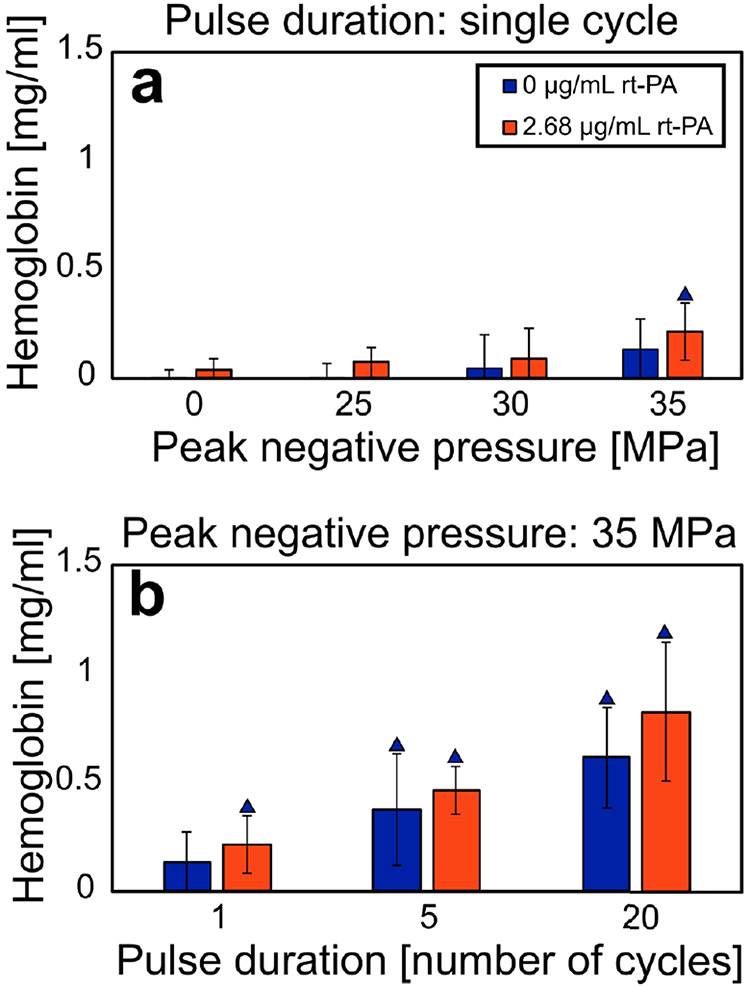
Clot mass loss for each experimental arm. Each experimental arm comprises at least eight samples. Error bars represent standard deviations. (a) Mass loss as a function of peak negative pressure for single-cycle pulses. (b) Mass loss as a function of pulse duration for 35-MPa peak negative pressure pulses. Orange (blue) triangles denote a significant difference from control arms at 0-MPa peak negative pressure with (without) lytic. Single-, five-, and twenty-cycle pulses correspond to 0.67-, 3.33-, and 13.3-*μ*s pulse duration, respectively.

The clot mass loss was used as the overall metric of treatment efficacy and is reported in Fig. 3. The relationship between the clot mass loss and the peak negative pressure of single-cycle pulses is shown in Fig. 3(a), and the relationship between pulse duration and clot mass loss is shown in Fig. 3(b). Trends indicating increase in clot mass loss were observed with increasing peak negative pressure, pulse duration, or lytic concentration. Overall, clots exposed to the combination of rt-PA and peak negative pressures of 30 MPa or greater resulted in significantly more mass loss than clots exposed to lytic alone (i.e., expected treatment efficacy with current standard of care).

### B. Quantification of Hemolysis and Fibrinolysis

**Fig. 4.**
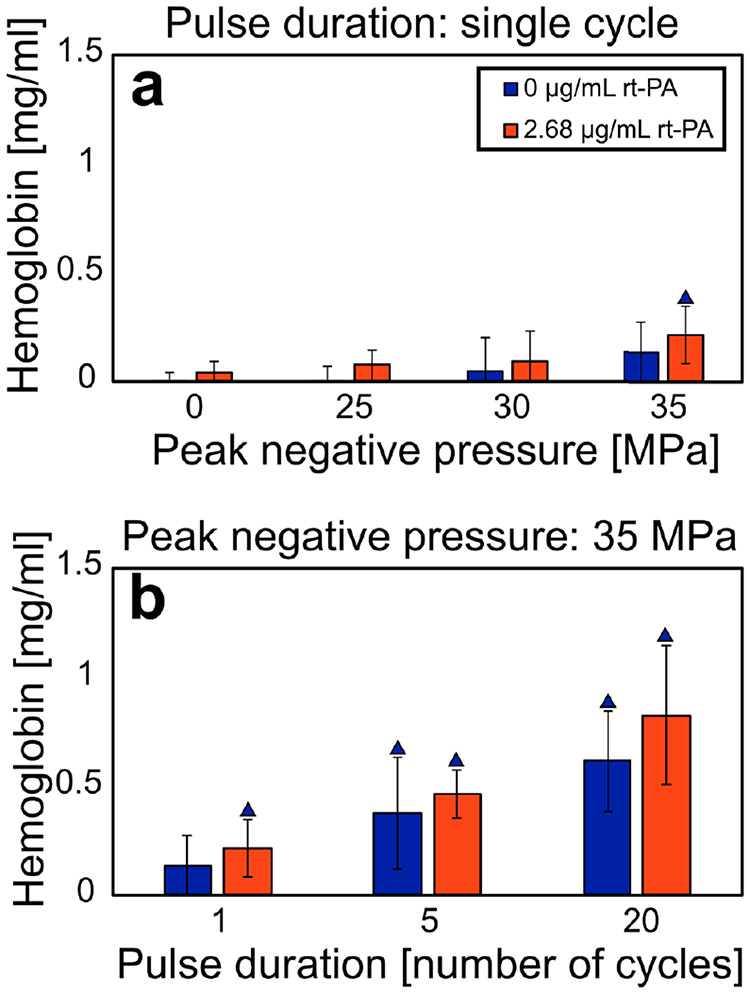
Hemoglobin concentration for each experimental arm indicating the degree of hemolysis. Each experimental arm comprises at least eight samples. Error bars represent standard deviations. (a) Hemoglobin as a function of peak negative pressure of the histotripsy pulse (single-cycle pulse duration only). (b) Hemoglobin as a function of the histotripsy pulse duration (35-MPa peak negative pressure only). Blue triangles denote a significant difference from control arms at 0-MPa peak negative pressure without lytic. For each insonation condition, the addition of rt-PA did not significantly change the amount of free hemoglobin. Single-, five-, and twenty-cycle pulses correspond to 0.67-, 3.33-, and 13.3-*μ*s pulse duration, respectively.

For each histotripsy insonation scheme, the resultant hemoglobin concentration was not statistically different for arms with and without lytic. These results indicate that rt-PA does not result in hemolysis and that free hemoglobin can be used a metric of the mechanical effects of histotripsy. The magnitude of clot fractionation increased with increasing peak negative pressure and pulse duration (see Fig. 4).

**Fig. 5.**
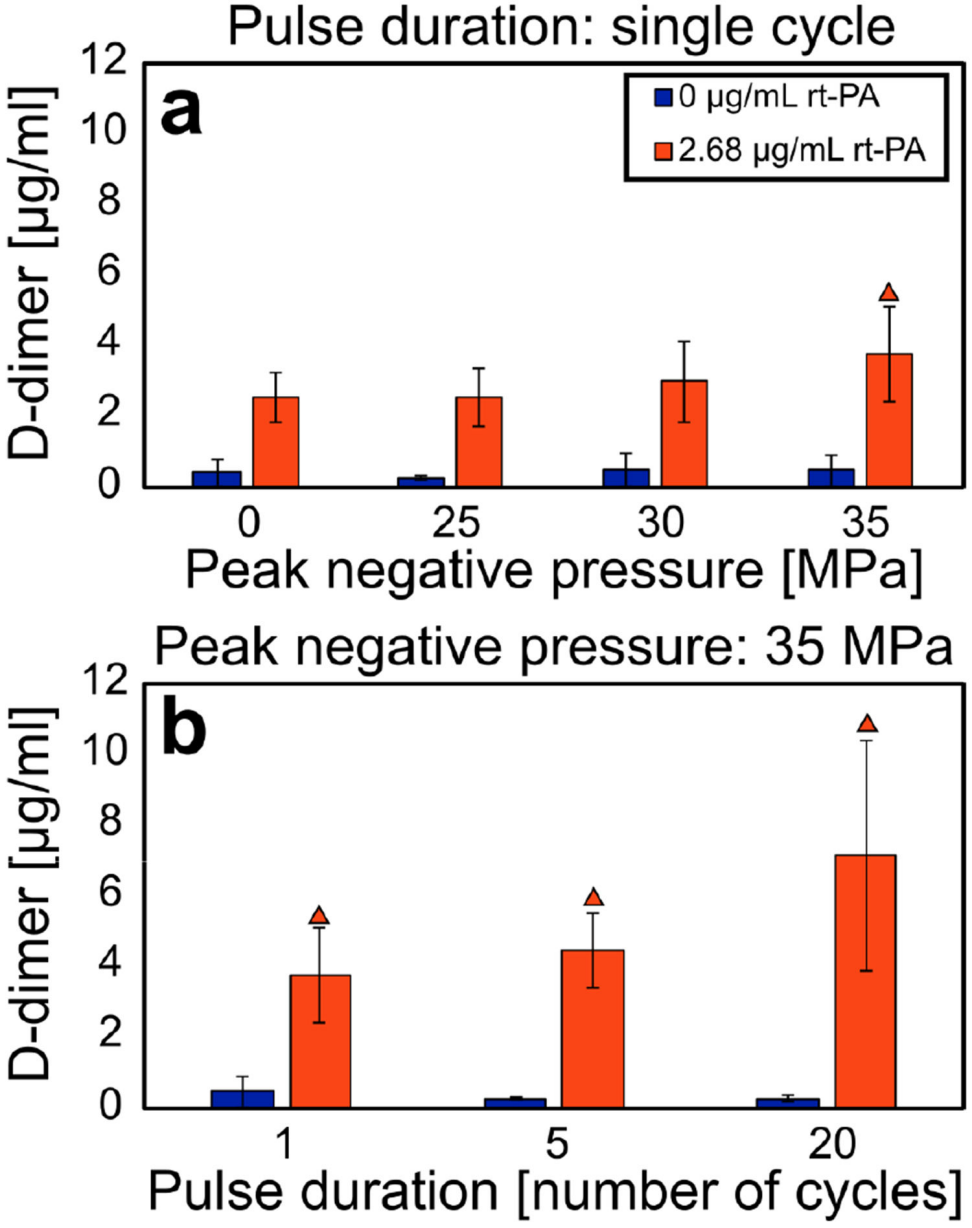
D-dimer concentration for each experimental arm. Each experimental arm comprises at least eight samples. Error bars represent standard deviations. (a) D-dimer as a function of peak negative pressure. (b) D-dimer as a function of cycles per pulse. Orange triangles denote a significant difference from control arms at 0-MPa peak negative pressure with lytic. For each histotripsy insonation condition, the addition of lytic significantly increases the D-dimer concentration in the perfusate compared to arms without rt-PA. Single-, five-, and twenty-cycle pulses correspond to 0.67-, 3.33-, and 13.3*μ*s pulse durations, respectively.

To quantify the fibrinolytic component of clot degradation, D-dimer was measured. As shown in Fig. 5, histotripsy in the absence of rt-PA generated D-dimer concentrations equivalent to clots exposed to plasma alone (no rt-PA and no histotripsy insonation). These data show that histotripsy insonation does not produce D-dimer directly from mechanical fractionation. Therefore, D-dimer can be used to quantify the contribution of rt-PA fibrinolysis to overall treatment efficacy. Increased fibrinolysis was observed for clots treated with rt-PA and histotripsy compared to plasma alone (see Fig. 5). Increased D-Dimer compared to rt-PA alone (clinical standard) was observed for histotripsy arms with 35-MPa peak negative pressures pulses combined with lytic.

Linear regression analysis was performed to assess the relationship between hemoglobin, D-dimer, and clot mass loss.

**Fig. 6.**
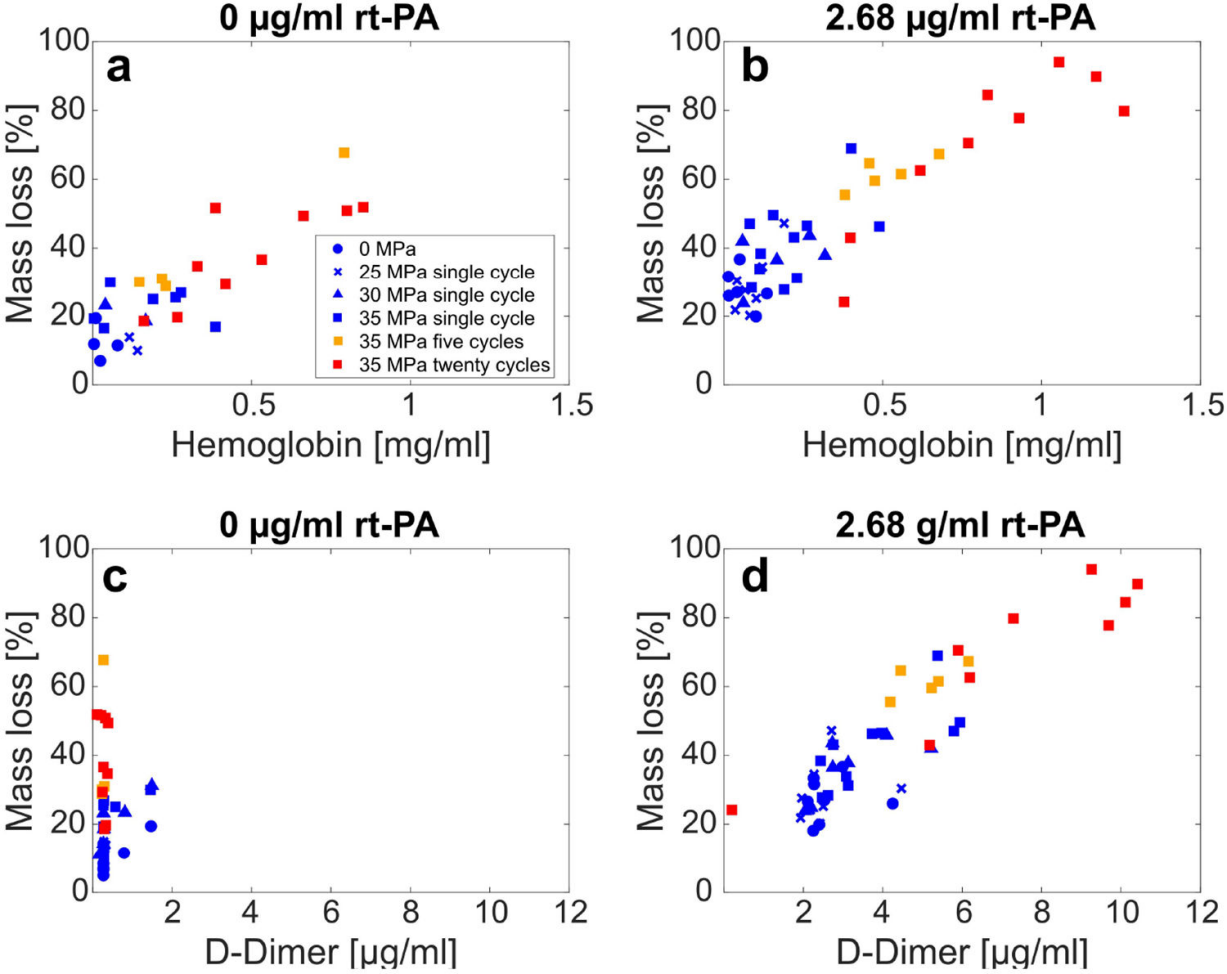
For all histotripsy insonation conditions, the relationship between (a) clot mass loss and hemoglobin without lytic, (b) clot mass loss and hemoglobin with lytic, (c) clot mass loss and D-dimer without lytic, and (d) clot mass loss and D-dimer with lytic. The legend in (a) indicating the histotripsy insonation parameters applies to all subfigures.

Standard score (z-score) values were computed for hemoglobin and D-dimer concentrations in order to compare the change of each variable relative to the change in mass loss directly. As a result, these normalized values are unitless, and the slopes of the linear regression have units of percent mass loss [%]. The change was quantified by the slope *β_M–X_*, where the subscript *M* refers to mass loss and *X* refers to the paired variable for regression analysis (H for hemoglobin and D for D-dimer). In the absence of lytic, *β_M–H_* = 13.6% [11.4%, 15.8%] [see Fig. 6(a)], and *β_M–D_* = 3.6% [1.2%, 5.6%] [see Fig. 6(c)]. Bracketed values denote the 95% confidence intervals of the slope, indicating a significant difference between *β_M–H_* and *β_M–D_* in the absence of rt-PA. For clots exposed to lytic, *β_M–H_* = 9.5% [6.0%, 13.0%] [see Fig. 6(b)] and *β_M–D_* = 10.4% [6.9%, 13.8%] [see Fig. 6(d)]. The slopes *β_M–H_* and *β_M–D_* calculated in the presence of lytic were not significantly different. Note that raw values for the concentration of hemoglobin and D-dimer are presented in Fig. 6, not z-scores.

### C. Bubble Cloud Emissions

**Fig. 7.**
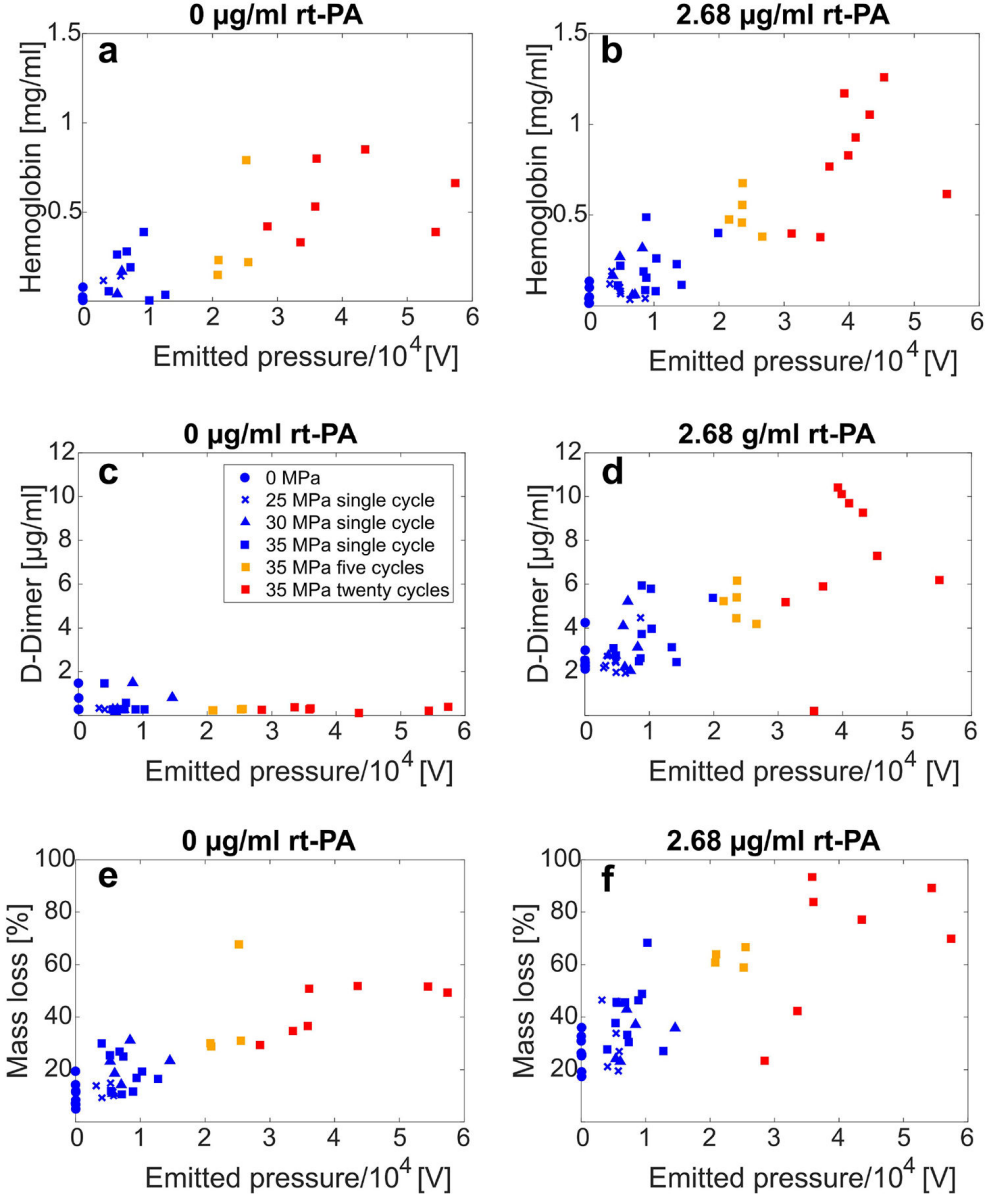
Relationship between acoustic emissions tracked with passive cavitation imaging and (a) hemoglobin for clots not exposed to lytic, (b) hemoglobin for clots exposed to lytic, (c) D-dimer for clots not exposed to lytic, (d) D-dimer for clots exposed to lytic, (e) mass loss for clots not exposed to lytic, and (f) mass loss for clots exposed to lytic. Histotripsy exposure conditions indicated in (c) apply for all subfigures.

A secondary aim of this study was to gauge the contribution of histotripsy bubble cloud activity to hemolysis and fibrinolysis. For each experiment, the tabulated acoustic emissions generated by the oscillations of the bubble cloud are compared to hemoglobin and D-dimer production in Fig. 7. The acoustic emissions are the coherent summation of the pressure field originating from histotripsy bubble activity incident on the imaging array [24], [43]-[45]. These emissions induce voltages across each element of the imaging array in passive receive mode and are reported in units of volts. The reported voltage signal is proportional to the pressure incident on the imaging array and is the coherent summation of the bubble cloud acoustic emissions [46]. A linear regression analysis was used to compute the slopes of hemoglobin with acoustic emissions (*β_H–A_*), and D-dimer with acoustic emissions (*β_D–A_*). Standard score (z-score) values were computed for hemoglobin and D-dimer values prior to computing *β*. Acoustic emissions are reported in units of volts [V], and therefore *β_H–A_* and *β_D–A_* have units of V^–1^. For treatment arms without lytic, *β_H–A_* = 0.051 V^−1^ [0.039, 0.064] V^−1^ [see Fig. 7(a)] and *β_D–A_* = −0.013 V^−1^ [−0.034, 0.008] V^−1^ [see Fig. 7(c)]. The 95% confidence interval of *β_D–A_* includes zero, indicating the majority of bubble cloud activity contributed to hemolysis in the absence of rt-PA. The slope between mass loss and acoustic emissions was also computed and found to be *β_M–A_* = 0.81 $\frac{\%}{V}$ [0.63, 0.99]$\frac{\%}{V}$[see Fig. 7(e)]. For clots exposed to lytic, *β_H–A_* = 0.059 V^−1^ [0.049, 0.069] V^−1^ [see Fig. 7(b)], and *β_D-A_* = 0.050 V^−1^ [0.036, 0.063] V^−1^ [see Fig. 7(d)]. The *β_H–A_* and *β_D–A_* values were statistically not different. For lytic arms, *β_M–A_* = 1·1 $\frac{\%}{V}$ [0.83, 1.32] $\frac{\%}{V}$ [see Fig. 7(f)].

### D. Histological Analysis

**Fig. 8.**
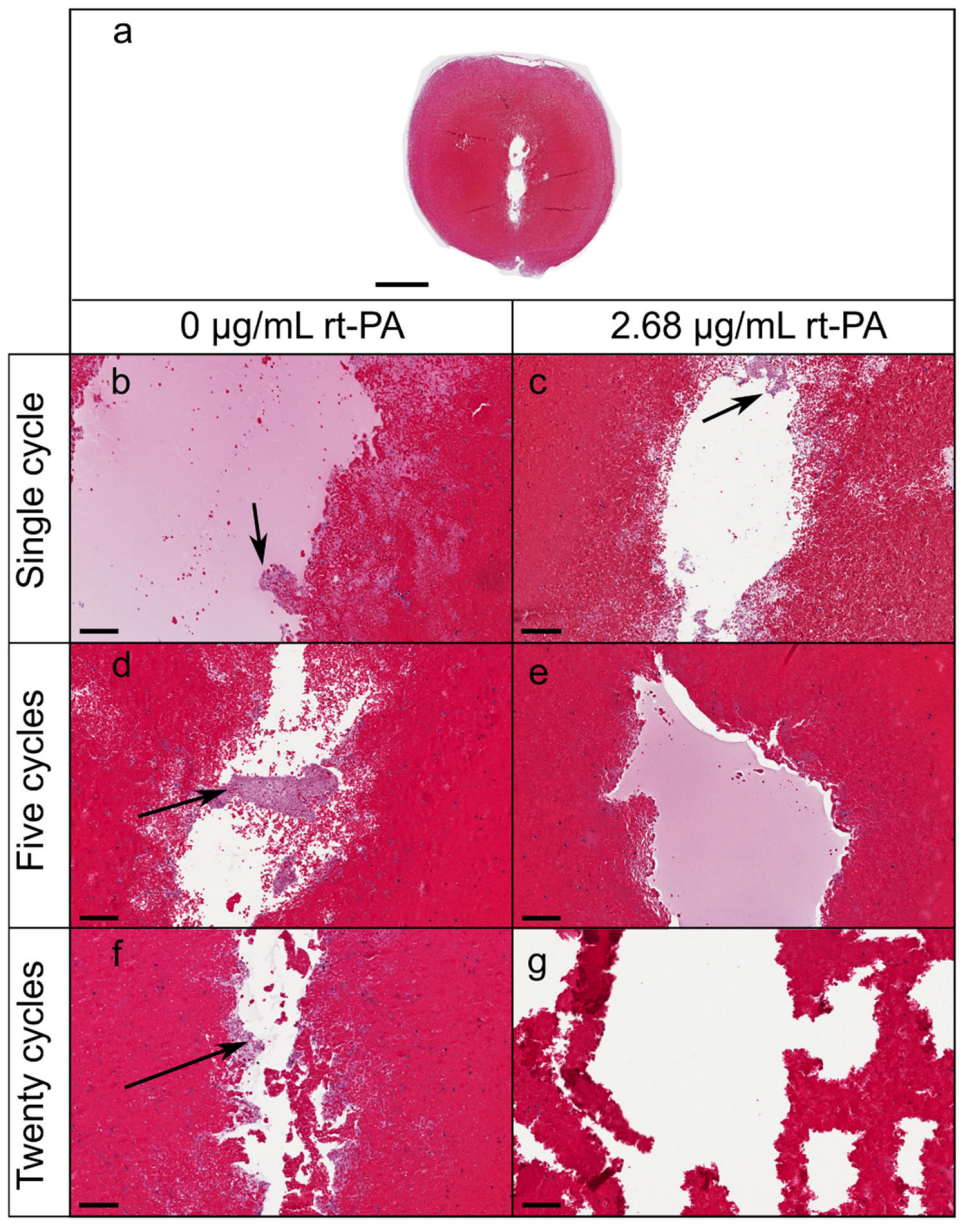
Representative histological sections of clots exposed to varying pulse durations (35 MPa) with and without lytic. In all cases, the histotripsy pulse propagated from the top to the bottom of the image. (a) Low-magnification image of a clot exposed to lytic and single-cycle pulses (scale bar denotes 1 mm). Damage was contained almost entirely within the clot. (b)–(g) High-magnification images of clots posttreatment (scale bar denotes 100 μm). Black arrows point to residual fibrin clusters. Generally, clots exposed to histotripsy alone contained more fibrin within the treatment zone in comparison to clots exposed to histotripsy and lytic.

To ascertain the qualitative effect of histotripsy and lytic on clot structure, samples were carefully removed from the flow channel and sectioned and stained with Hematoxylin & Eosin. Damage to clot was primarily restricted to the center of the structure, as indicated by a reduction in red blood cells and fibrin mesh [see Fig. 8(a)]. A sharp separation (less than 40 *μ*m) was observed between intact and ablated portions of the clot. Smaller fibrin clusters were observed at the edge of the treatment zone for clots exposed to histotripsy and rt-PA compared to histotripsy alone (see Fig. 8). The reduced size and limited presence of the fibrin clusters were attributed to action of lytic. For the treatment arms with rt-PA, the extent of fibrin near the treatment zone decreased as the pulse duration increased, with no observable fibrin for the 20 cycle pulses. Though significant damage was observed within the center of the clot, the fibrin structure along the perimeter of the damage zone appeared unaffected by the histotripsy insonation parameters for arms without lytic.

## IV. Conclusion and Discussion

### A. Quantification of Clot Dissolution

Previous studies have demonstrated effective clot dissolution when combining histotripsy bubble cloud activity with a lytic therapy [15], [24]. Here, the nature of clot degradation under this combination therapy, and how bubble cloud oscillations contribute to hemolysis and fibrinolysis, was investigated. Hemolysis was a contributing factor for all insonation schemes [see Fig. 6(a) and (b)] and was the primary contributor to mass loss in the absence of lytic [see Fig. 6(a) and (c)]. Erythrocyte disruption would be expected based on the well-established correlation between histotripsy and tissue ablation at the cellular level [47]. Interestingly, *β_M–H_* (the slope comparing changes in hemoglobin to changes in mass loss) was not significantly different for arms with and without rt-PA, indicating the contribution of hemolysis to mass loss occurred independently of any concurrent fibrinolysis.

A strong increase was noted for the slope between D-dimer and mass loss, *β_M–H_*, for arms with lytic compared to without lytic [see Fig. 6(c) and (d)]. Furthermore, almost no D-dimer was detected for arms without rt-PA. Histological analysis of the clots following histotripsy exposure demonstrated significant disruption of the fibrin network at the center of the ablation zone for all arms, including clots not exposed to lytic (see Fig. 8). These results indicate that though the fibrin network is damaged by histotripsy exposure, it is not reduced to D-dimer by histotripsy alone. In the presence of lytic, histotripsy may contribute to fibrinolysis as indicated by the production of D-dimer in several ways. Fluid mixing induced by bubble oscillations may increase rt-PA penetration into the clot [31]. Maxwell *et al.* [12] have previously shown that cavitation can enhance streaming and generate vortices. Bubble cloud-induced vortices in the perfusate may entrain lytic and plasminogen within the clot, thereby increasing the likelihood of interaction with the fibrin mesh. Mechanical fractionation will erode and alter the clot shape, including the potential formation of channels within its structure (see Fig. 8). This damage will increase the surface area exposed to a lytic relative to the volume, promoting favorable lytic-to-clot interactions.

Clot mass loss may also occur through detachment of intact erythrocytes from the clot. Histotripsy and lytic both weaken the fibrin mesh, which can lead to the shedding of erythrocytes. Although intact erythrocytes were not analyzed in this study, numerous studies have demonstrated that tissue debris following histotripsy exposure is primarily subcellular in size [15], [24], [26], [48].

A further finding of note was the statistical equivalence between *β_M–H_* and *β_M–D_* for arms combining histotripsy and rt-PA. The equivalence of these slopes indicates that increasing hemoglobin by one standard deviation yielded the same mass loss as increasing D-dimer by one standard deviation, that is, hemolysis and fibrinolysis contribute equally to the overall thrombolytic efficacy in the presence of lytic and are both critical for the success of this combination therapy. This finding may be specific to the clot model used in these studies, which is rich in both fibrin and erythrocytes. As a thrombus ages *in vivo*, the cellular structure is gradually replaced with more extensive extracellular components besides fibrin [11]. For such samples, fibrinolysis and hemolysis may have a reduced effect on treatment efficacy due to the thrombus composition. It should also be noted that 2000 histotripsy pulses were applied at each location in the clot, which likely resulted in overtreatment [20]. Therefore, the relative contribution of hemolysis and fibrinolysis to overall treatment efficacy may shift upon the development of future insonation schemes that avoid overtreatment. Regardless, hemolysis and fibrinolysis are both significant contributors to the efficacy of this approach.

### B. Influence of Insonation Parameters on Hemolysis and Fibrinolysis

Over the range of insonation parameters explored here, the duration of the histotripsy pulse had a stronger influence on clot dissolution metrics (mass loss, hemoglobin, and D-dimer) compared to the peak negative pressure. This result may in part be due to the volume of the bubble cloud, and the relative proximity between bubbles and rt-PA. The influence of peak negative pressure was investigated for single-cycle histotripsy pulses, an approach known to produce very precise, small ablation zones within the clot [26]. Preliminary qualitative analysis of bubble cloud localization based on hyperechogenicity with B-mode imaging indicated confinement of the bubble cloud within the clot for single-cycle pulses. The actual degree of hemoglobin generated may be underestimated for highly localized bubble clouds if these products are generated in the center of the clot and unable to diffuse through viable portions of the clot into the perfusate. Lytic was infused in the plasma surrounding the clot and was thus not in close proximity to bubble cloud activity contained within the center of the clot for single-cycle pulses. In contrast, bubble clouds generated by multicycle pulses encompassed a larger portion of the lumen (see Fig. 2), resulting in robust degrees of hemolysis and fibrinolysis. These observations may indicate the need for bubble activity to be in close proximity to the lytic to promote fibrinolysis. The lytic infusion scheme used in this study mimics the administration of systemic rt-PA. In contrast, catheter-directed thrombolytics are the preferred treatment method for iliofemoral venous thrombosis [49], [50], which may promote strong fibrinolysis with single-cycle histotripsy pulsing schemes.

### C. Bubble Cloud Emissions

A secondary objective of this study was to assess the contribution of histotripsy-induced bubble cloud activity to hemolysis and fibrinolysis. Passive cavitation imaging was used to track the acoustic emissions, a surrogate for the degree of mechanical activity. Previous studies have demonstrated a strong relationship between bubble activity tracked with passive cavitation imaging and hemolysis [51], [52]. The data collected here indicate the change in hemolysis with bubble activity (*β_H–A_*) was not different for arms with and without rt-PA. Hence, the presence of rt-PA did not influence erythrocyte degradation under the action of histotripsy bubble cloud activity.

Similar to the contributions to mass loss, the presence of rt-PA was necessary for bubble activity to promote fibrinolysis (i.e., *β_D–A_* > 0). Prior studies have established that acoustic emissions can be used to gauge overall thrombolytic efficacy [24], hemolysis [53], or histotripsy ablation [43]-[45]. Our data extend upon those previous findings to demonstrate a direct connection between acoustic emissions and rt-PA activity to promote fibrinolysis. The contributions of bubble activity to hemolysis and fibrinolysis in the presence of rt-PA, reported by the slopes *β_H–A_* and *β_D–A_*, respectively, were statistically equivalent. It should be noted that bubble activity depends nonlinearly on the type and concentration of bubble nuclei and medium [54], and on the insonation scheme [24]. Therefore, the findings here related to bubble contributions to overall treatment efficacy may not extend to other histotripsy-based treatment schemes.

### D. Limitations

There are several aspects of this *in vitro* study that limit the generalizability of these findings. The primary metric of fibrinolytic activity in this study was D-dimer. The interaction between rt-PA and fibrin was assumed to occur in close proximity to the clot. It is possible that histotripsy bubble activity released fibrin strands from the clot that subsequently interacted with lytic. Degradation of released fibrin strands via rt-PA would result in an overestimate of the overall fibrinolytic contribution to clot mass loss. The contribution of histotripsy bubble activity in generating ejected fibrin strands will be the focus of future studies. A partially occlusive clot model was employed in this study, whereas there would be limited lytic delivery for total vascular occlusion with the administration scheme employed in this study. The flow rate was fixed and neglected the contribution of increased flow rate and shear stress as the clot lyses [55]. Retracted human whole-blood clots were employed in this study, whereas several thrombus phenotypes have been identified in vascular occlusions [11], [56]. Though clot mass loss was the primary metric of treatment efficacy in this study, recanalization (i.e., flow) is the primary clinical metric of treatment efficacy for venous thromboembolism [57], [58]. Overall, these results indicate that histotripsy is a useful adjuvant therapy that enhances the current standard of care (lytic therapy) while providing the benefit of an effective ablative therapy.

### E. Summary

For the acoustic parameters considered here, we have shown that clot degradation in the presence of rt-PA is caused by fibrinolysis and hemolysis. In addition, we have shown that histotripsy bubble activity contributes equally to fibrinolysis and hemolysis. These results indicate that histotripsy is a promising adjuvant to thrombolytic therapy.
